# Hypoxia accelerates vascular repair of endothelial colony-forming cells on ischemic injury via STAT3-BCL3 axis

**DOI:** 10.1186/s13287-015-0128-8

**Published:** 2015-07-29

**Authors:** Sang Hun Lee, Jun Hee Lee, Yong-Seok Han, Jung Min Ryu, Yeo Min Yoon, Ho Jae Han

**Affiliations:** Medical Science Research Institute, Soonchunhyang University Seoul Hospital, Seoul, 140-743 Republic of Korea; Department of Biochemistry, Soonchunhyang University College of Medicine, Cheonan, 330-930 Republic of Korea; Laboratory for Vascular Medicine & Stem Cell Biology, Medical Research Institute, Department of Physiology, School of Medicine, Pusan National University, Yangsan, 626-870 Republic of Korea; Department of Veterinary Physiology, College of Veterinary Medicine, Research Institute for Veterinary Science, and BK21 PLUS Creative Veterinary Research Center, Seoul National University, Seoul, 151-742 Republic of Korea

## Abstract

**Introduction:**

Endothelial colony-forming cells (ECFCs) significantly improve tissue repair by providing regeneration potential within injured cardiovascular tissue. However, ECFC transplantation into ischemic tissue exhibits limited therapeutic efficacy due to poor engraftment in vivo. We established an adequate ex vivo expansion protocol and identified novel modulators that enhance functional bioactivities of ECFCs.

**Methods:**

To augment the regenerative potential of ECFCs, functional bioactivities of hypoxia-preconditioned ECFCs (hypo-ECFCs) were examined.

**Results:**

Phosphorylations of the JAK2/STAT3 pathway and clonogenic proliferation were enhanced by short-term ECFC culturing under hypoxia, whereas siRNA-targeting of STAT3 significantly reduced these activities. Expression of BCL3, a target molecule of STAT3, was increased in hypo-ECFCs. Moreover, siRNA inhibition of BCL3 markedly reduced survival of ECFCs during hypoxic stress in vitro and ischemic stress in vivo. In a hindlimb ischemia model of ischemia, hypo-ECFC transplantation enhanced blood flow ratio, capillary density, transplanted cell proliferation and survival, and angiogenic cytokine secretion at ischemic sites.

**Conclusions:**

Hypoxia preconditioning facilitates functional bioactivities of ECFCs by mediating regulation of the STAT3-BCL3 axis. Thus, a hypoxic preconditioned ex vivo expansion protocol triggers expansion and functional bioactivities of ECFCs via modulation of the hypoxia-induced STAT3-BCL3 axis, suggesting that hypo-ECFCs offer a therapeutic strategy for accelerated neovasculogenesis in ischemic diseases.

## Introduction

Patients with peripheral arterial disease are at risk for progression to severe limb ischemia. Therapeutic angiogenesis is important for blood perfusion in ischemic limb tissue and tissue regeneration after critical ischemia [1, 2]. Stem cell-based therapy holds great promise for therapeutic angiogenesis in ischemic limb diseases [3]. Circulating endothelial progenitor cells (EPCs), an angiogenesis potential-initiating subpopulation, were originally identified in adult peripheral blood, and bone marrow (BM)-derived stem/progenitor cells are required for several activities of EPCs. EPCs have the ability to self-renew in the BM, differentiate into mature endothelial cells, and then mobilize from the BM to the circulatory system. Furthermore, they are recruited to sites of neovascularization [4]. Accumulating evidence suggests that transplantation of human circulating EPCs enhances vascular repair and regeneration following ischemic diseases [5, 6].

Hypoxia is known to regulate cellular processes and signal transduction via the expression of hypoxia inducible factor-1α (HIF-1α), which is regulated by cellular O_2_ concentration and determines the transcriptional activity of HIF-1 [7]. HIF-1α exerts significant effects on the bioactivities of both cancer and stem cells by stimulating cell proliferation, vascular endothelial growth factor (VEGF) expression, and angiogenesis [8, 9]. The activity and stability of HIF-1α are known to be modulated by STAT3. Activated STAT3 increases HIF-1α protein levels and stability by accelerating de novo synthesis and blocking degradation [10]. Pawlus et al. [11] demonstrated that STAT3 specifically binds to the promoters of HIF-1 and HIF-2 target genes, interacting with HIF-1α to activate HIF-1 target gene promoters, even when overexpressed. Recently, the relationship between STAT3 and BCL3 has been demonstrated in carcinoma and tumor survival [12]. Furthermore, BCL3 has been suggested to be involved in the pathogenesis of solid tumors such as nasopharyngeal carcinoma [13] and breast cancer [14]. Stem and cancer cells share many similarities in gene expression, cellular processes, and signal transduction pathways, but few, if any, studies have evaluated the effects of the STAT3-BCL3 axis in normal stem cells. In addition, it is not clear whether hypoxic culture is beneficial to each type of stem cell owing to their various origins and differences in oxygen sensitivity [15].

After localization to ischemic tissue, EPCs encounter severe hypoxic conditions, ranging from 0.4 to 2.3 % O_2_, often resulting in apoptosis [16]. However, before exposure to severe conditions at the site of ischemic injury, preconditioning of cells in less severe hypoxic conditions (1–3 % O_2_) is able to circumvent hypoxia-induced apoptosis through induction of p42/44 mitogen-activated protein kinases [17]. Previous studies have shown that culture in hypoxic conditions (2–7 % O_2_) is beneficial for EPCs, as this oxygen tension is similar to that in the physiological niche for EPCs in the BM; it maintains their viability and enhances the proliferation rate of mesenchymal stem cells (MSCs) via expression of fibronectin [18], collagen I [19], connexin 43 [19], CXCR4 [20] and angiogenic cytokines [21]. In this study, we hypothesized that hypoxic culture would provide additional benefits to endothelial colony-forming cells (ECFCs) over normoxic culture; thus, we attempted to establish a general protocol for the high proliferation of ECFCs within a hypoxic culture. Specifically, we determined the effects of hypoxic preconditioned culture on the clonogenic and proliferative potential of ECFCs, as well as on their ability to promote therapeutic vasculogenesis in ischemic limbs. Furthermore, we investigated the effects of hypoxic preconditioning on ECFC proliferation, survival, and VEGF secretion via regulation of the BCL3-STAT3 circuit.

## Methods

### Animal

Experiments were performed on male 8-week-old Balb/C nude mice (Orient Bio, Seoul, Korea [22]) maintained under a 12-h light/dark cycle and in accordance with the regulations of Soonchunhyang University. All procedures were approved by guidelines of the Institutional Animal Care and Use Committee of Soonchunhyang University, Seoul Hospital, Korea (IACUC2013-5).

### Isolation, cell culture, and characterization of ECFCs

Isolation and culture of ECFCs was performed according to previously described methods, but with minor modifications [23]. Human umbilical cord blood (HUCB) was collected from three healthy volunteers after obtaining informed consent from all subjects according to the protocol approved by the Institutional Review Board of the Pusan National University Yangsan Hospital, Korea (IRB No. 2012–19). HUCB samples were collected from fresh placentas with attached umbilical cords. Human mononuclear cells (MNCs) were obtained as previously described, with minor modifications [24–26]. MNCs were isolated from HUCB by density gradient centrifugation with a Ficoll separating solution (Amersham Biosciences, Uppsala, Sweden). Freshly isolated MNCs were cultured on 100-mm dishes coated with 1 % gelatin (Sigma, St. Louis, MO, USA) and cultured in endothelial basal media (EBM)-2 (Lonza, Walkersville, MD, USA) supplemented with 5 % fetal bovine serum (FBS), human VEGF, human basic fibroblast growth factor (bFGF), human epidermal growth factor (EGF), human insulin-like growth factor-1 (IGF-1), ascorbic acid, and GA-1000 (complete EGM-2 medium). After 4 days, nonadherent cells were discarded and fresh culture medium was applied. Cultures were maintained for another 3 days, and extended culture was continued until the appearance of spindle-shaped colonies (~14–21 days) by refreshing with fresh EGM-2 media. The medium was changed daily for 7 days, and then every 2 days until the first passage. The ECFC (passage 6) population was determined via flow cytometry analysis using labeled endothelial cell markers (i.e., anti-human CD31 (PECAM-1; BD Pharmingen, San Diego, CA, USA), anti-human CD144 (VE-cadherin; BD Pharmingen), anti-human von Willebrand factor (vWF; Santa Cruz Biotechnology, Dallas, TX, USA), and anti-human Tie-2 (BD Pharmingen)). Stained cells were analyzed using a fluorescence-activated cell sorter (FACS; BD FACS Canto II, San Jose, CA, USA). Flow cytometry analysis was performed according to previously described methods with minor modifications [27]. For FACS analysis, soon after hypoxic preconditioning, we performed fixation for 24 h to minimize reperfusion stress. ECFCs expressed high levels of the endothelial cell markers CD31, CD144, vWF, and Tie-2 following expansion under both normoxic and hypoxic conditions for 24 h. Each experiment was repeated at least three times.

### Hypoxia and hypoxic preconditioning

To study the effect of hypoxia or hypoxic preconditioning on ECFCs, ECFCs were incubated in a Modular Incubator Chamber (IB Science, Daejeon, Korea) that maintained a gas mixture composed of 93 % N_2_, 5 % CO_2_, and 2 % O_2_. Hypoxic preconditioning was performed by incubating cells for 24 h at 37 °C under hypoxic conditions (2 % O_2_).

### Single-cell assays

Flow cytometry (BD FACS Aria II) was used to place one single ECFC into a well of a 96-well flat-bottom tissue culture plate precoated with 1 % gelatin and containing 200 μL complete EGM-2 media. Individual wells were examined with an inverted phase contrast microscope at 40× magnification to ensure that only one cell had been placed into each well. Cells were cultured under normoxic (94.5 % air and 5.5 % CO_2_) or hypoxic (93 % N_2_, 5 % CO_2_, and 2 % O_2_) conditions in a humidified incubator. At day 10, each well was examined for ECFC growth. To assess the clonogenicity of single ECFCs, we used phase contrast microscopy to score all colony-forming wells per 96-well plate. To enumerate the number of cells per colony-forming well, we trypsinized colony-forming cells and counted them with a hemocytometer [23].

### Intracellular accumulation of calcein AM

Single ECFCs were placed into the wells of a 96-well flat-bottom tissue culture plate and incubated with normoxia or hypoxia preconditioning for 10 days. Subsequently, 50 μL of 1 μM calcein AM (Invitrogen, Carlsbad, CA, USA) was added to 100 μL of the culture medium, which was contained in each microplate well. After incubation with calcein AM for 30 min at 37 °C, the medium was removed by aspiration. The intracellular accumulation of calcein AM was then visualized and quantified by using a fluorescence microscope (Nikon, Tokyo, Japan).

### Small interfering RNA transfection of cells

ECFCs were grown to 75 % confluency and then transfected for 24 h with SMART pool small interfering RNAs (siRNAs) specific to either STAT3 or BCL3 (both at 100 nM) using Lipofectamine 2000 (Invitrogen) according to the manufacturer’s instructions.

### Western blot analysis

Total protein was extracted using RIPA Lysis Buffer (Thermo Scientific, Rockford, IL, USA). Cell lysates were separated by sodium dodecyl sulfate-polyacrylamide gel electrophoresis, and proteins were transferred to polyvinylidene fluoride membranes (PVDF; Millipore, Billerica, MA, USA). The membranes were blocked with 5 % skim milk and incubated with primary antibodies against human-specific BCL3, cleaved caspase-3, human VEGF, STAT3, phospho-STAT3, α-tubulin, and β-actin (Santa Cruz Biotechnology). After incubation of the membranes with peroxidase-conjugated secondary antibodies (Santa Cruz Biotechnology), bands were visualized using enhanced chemiluminescence (ECL) reagents (Amersham Biosciences).

### Human VEGF assay

Human VEGF concentrations in cell culture media were determined with the human VEGF enzyme-linked immunosorbent assay (ELISA) reagents (BD Pharmingen) according to the manufacturer’s instructions. Absorbance was read at 450 nm on a spectrophotometric ELISA plate reader. Results are expressed as the mean concentration of triplicate cultures. Values were converted from absolute counts to a percentage of the control to allow for comparison between experimental groups.

### Cell transplantation in a murine hindlimb ischemia model

In the murine hindlimb ischemia model, ischemia was induced by ligation of the proximal femoral artery and boundary vessels of the mice. No later than 6 h after surgery, phosphate-buffered saline (PBS), nor-ECFCs, hypo-ECFCs, si-STAT3 hypo-ECFCs, and si-BCL3 hypo-ECFCs were injected intramuscularly into the ischemic thigh area (5 × 10^5^ cells in 100 μL PBS per mouse, five mice per treatment group). Blood perfusion was assessed by measuring the ratio of the ischemic (left) limb blood flow/nonischemic (right) limb blood flow on postoperative days 0, 4, 9, 18, and 28 using laser Doppler perfusion imaging (LDPI; Moor Instruments, Wilmington, DE, USA).

### Histopathology and immunohistochemistry

After 3 and 28 days following ECFC transplantation, the ischemic thigh areas were removed and fixed with 4 % paraformaldehyde (Affymetrix, Santa Clara, CA, USA). Each tissue sample was embedded in paraffin. For histological analysis, samples were stained with hematoxylin and eosin (H&E). Immunofluorescence staining was performed using primary antibodies against mouse-specific (to confirm mouse vasculature) or human-specific (to confirm human ECFC-derived vessel formation) CD31, α-smooth muscle actin (α-SMA), human VEGF, phospho-STAT3, caspase-3, PCNA, Ki67 (Santa Cruz), human-specific CD31 (Novus Biologicals, Colorado, USA), and human nuclear antigen (HNA; Millipore) and secondary antibodies Alexa-488 and Alexa-594 (Life Technologies, Carlsbad, CA, USA). Nuclei were stained with 4′,6-diamidino-2-phenylindole phenylindole (DAPI; Vector Laboratories, Burlingame, CA, USA). Immunostained slides were imaged by confocal microscopy (Olympus, Japan).

### Hematoxylin staining

Single ECFCs in the wells of a 96-well flat-bottom tissue culture plate were incubated with normoxia or hypoxia preconditioning for 10 days, after which the ECFCs were washed twice in PBS and fixed in 4 % (w/v) paraformaldehyde in PBS for 40 min. The ECFCs were then washed twice more in PBS (15 min per wash) and subsequently immersed in Harris hematoxylin solution (Sigma) for 10 min. After a 20 min wash with PBS, the ECFC plates were photographed using a digital camera (Olympus).

### Statistical analyses

The results are expressed as mean ± SD. All experimental results were analyzed by analysis of varaince, and in some cases were followed by a comparison of the treatment means with the control using a Bonnferroni-Dunn test. Statistical significance was established at *P* values < 0.05.

## Results

### Characterization of ECFC surface markers in response to hypoxia

**Fig. 1:**
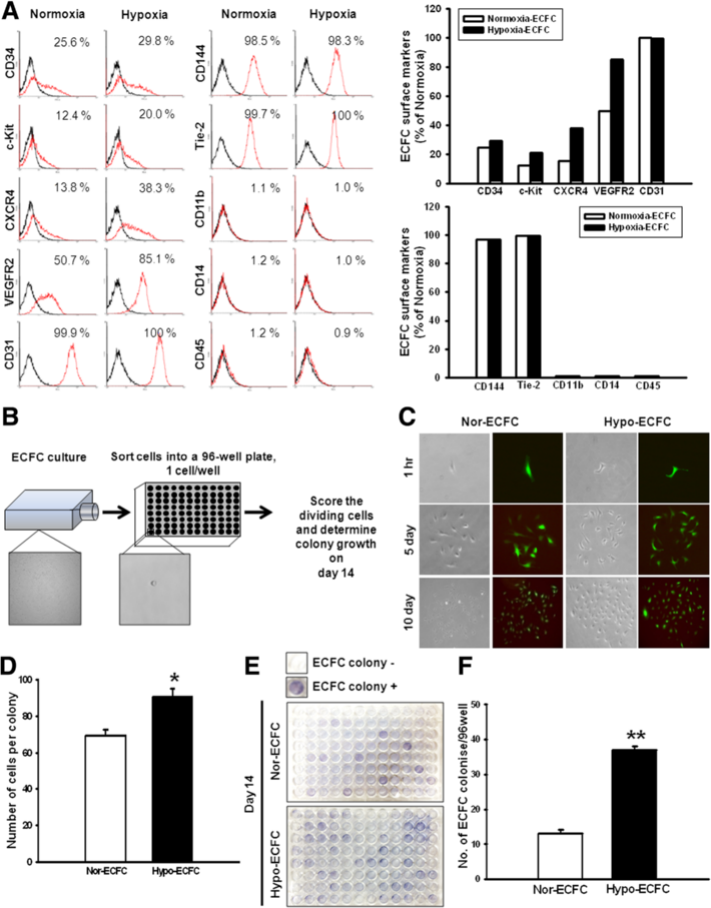
Enhanced proliferative and clonogenic potential of endothelial colony-forming cells (ECFCs) after hypoxic preconditioning. **a** Characterization of ECFC surface markers after hypoxic preconditioning for 24 h. FACS analysis was performed using various surface markers, including progenitor markers (CD34, c-Kit, and CXCR4), endothelial lineage markers (VEGFR2, CD31, CD144, and Tie-2), and hematopoietic markers (CD11b, CD14, and CD45). **b** Schematic of single-cell assays using ECFCs. **c** Representative photomicrograph of the cell clusters derived from a single ECFC. **d** The number of cells per nor-ECFC or hypo-ECFC colony that underwent at least one cell division after 10 days of culture (*P < 0.05 vs. nor-ECFCs). **e** Image of hematoxylin-stained plate with a representative ECFC colony, and **f** a graph of the number of ECFC colonies per 96-well plate. The example shown is representative of four independent experiments. **P < 0.01 vs. nor-ECFCs

To characterize surface markers of ECFCs in response to hypoxia, we established FACS analysis to assess various surface markers, including progenitor markers (CD34, c-Kit, and CXCR4), endothelial lineage markers (VEGFR2, CD31, CD144, and Tie-2), and hematopoietic lineage markers (CD11b, CD14, and CD45). The expression of progenitor markers and VEGFR2 was significantly increased in hypo-ECFCs compared with nor-ECFCs (Fig. 1a). Both hypo- and nor-ECFCs did not express hematopoietic lineage markers.

### Promotion of the proliferative and clonogenic potential of ECFCs in response to hypoxia

To investigate the effect of hypoxia on ECFCs, we developed an assay to quantify the proliferative and clonogenic potential of single cord blood derived from ECFCs (Fig. 1b). Remarkably, the average number of single cells undergoing at least one cell division was increased 1.3-fold for hypo-ECFCs compared with nor-ECFCs (Fig. 1c, d). In addition, the average number of ECFC colonies per plate of hypo-ECFCs was significantly greater than nor-ECFCs (Fig. 1e, f). These findings suggest that hypoxia influences the maintenance ability of ECFCs in vitro.

### Involvement of the JAK2/STAT3 pathway in hypoxic preconditioning of ECFC proliferative and clonogenic potential

**Fig. 2:**
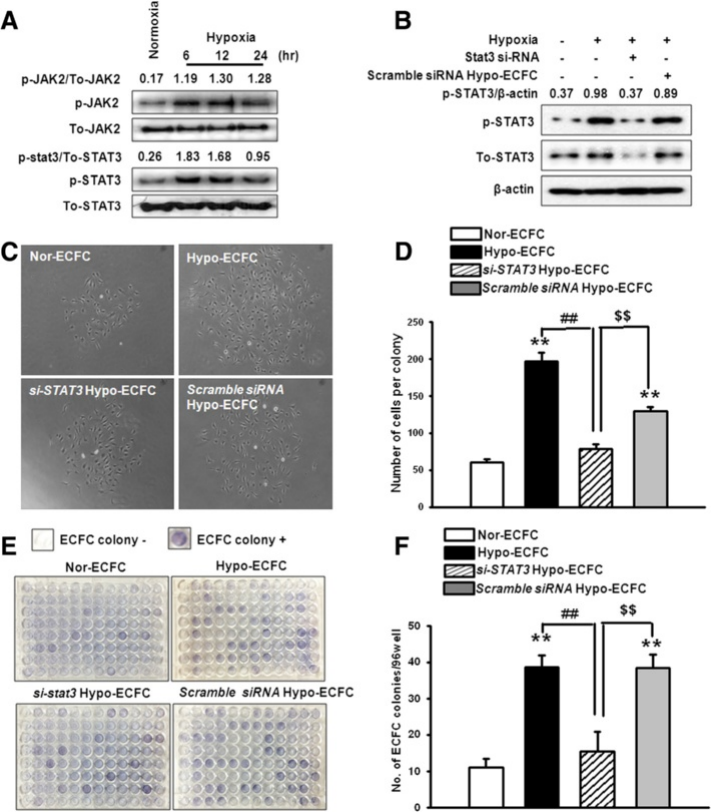
Activation of the JAK2/STAT3 signaling pathway in hypo-ECFCs and the regulation of proliferative, clonogenic potential. **a** Endothelial colony-forming cells (ECFCs) were exposed to hypoxia for 0–24 h, and JAK2, p-JAK2, STAT3, and p-STAT3 were detected by western blot analysis. **b** ECFCs were transfected with *STAT3*-siRNA and scramble siRNA for 48 h prior to hypoxia exposure for 24 h. p-STAT3 was detected by western blot analysis. **c** Representative photomicrograph of the cell clusters derived from single nor-ECFCs, hypo-ECFCs, *STAT3*-siRNA-transfected hypo-ECFCs (si-*STAT3*/hypo-ECFCs), and scramble siRNA-transfected hypo-ECFCs, as well as **d** a graph of the number of these cells per colony. **e** Images of hematoxylin-stained plates with representative nor-ECFC, hypo-ECFC, si-*STAT3*/hypo-ECFC, and scramble siRNA-transfected hypo-ECFCs colonies, as well as **f** graphs of the number of these colonies per 96-well plate. The example shown is representative of four independent experiments. **P < 0.01 vs. nor-ECFCs; ^##^ *P* < 0.01 vs. hypo-ECFCs; ^$$^ *P* < 0.01 vs. si-*STAT3*/hypo-ECFC

To assess the involvement of JAK2/STAT3 in hypoxic preconditioning of the proliferative and clonogenic potential of ECFCs, hypoxia-induced phosphorylation of JAK2 and STAT3 was examined by western blot. Hypoxia increased JAK2 and STAT3 phosphorylation in a time-dependent manner (Fig. 2a). We then examined whether STAT3 plays a positive role in the regulation of the proliferative and clonogenic potential in ECFC. The hypoxia-induced increase in STAT3 phosphorylation was attenuated by STAT3-specific siRNAs (Fig. 2b). Correspondingly, siRNA targeting of STAT3 in hypoxic cells decreased single-cell division as well as the number of colonies (Fig. 2c–f). This suggests that hypoxia increases proliferative and clonogenic potential through the activation of STAT3.

### Involvement of the STAT3 pathway in hypo-ECFC survival in ischemic tissue

**Fig. 3:**
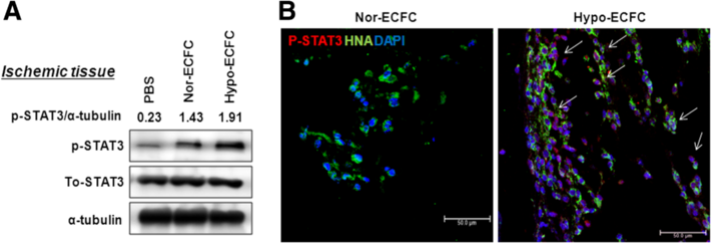
STAT3 is activated in hypo-ECFCs under ischemia. **a** Western blot analysis was performed to determine the levels of p-STAT3 in hindlimb ischemia injury after transplantation of hypo-ECFCs. **b** Co-immunofluorescence staining was used to detect p-STAT3 and hypo-ECFCs (human nuclear antigen (HNA)-positive cells, red). DAPI (blue) was used for nuclear staining. White color indicates merged color with green and red colors. *Arrows* indicate p-STAT3^+^/HNA^+^/DAPI^+^ cells. *ECFC* endothelial colony-forming cell, *PBS* phosphate-buffered saline

**Fig. 4:**
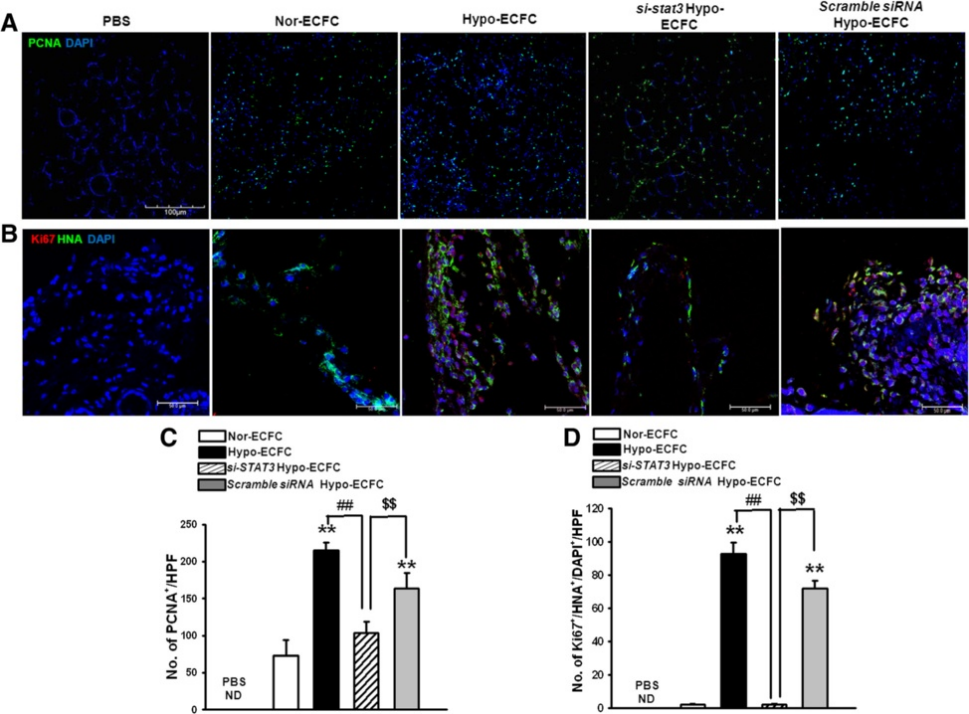
STAT3-mediated transplanted hypo-ECFC proliferation in ischemic tissues. **a** Immunofluorescence staining for proliferating cell nuclear antigen (PCNA, green) in ischemic hindlimb after transplantation of nor-ECFCs, hypo-ECFCs, *si-STAT3/*hypo-ECFCs, or scramble siRNA/hypo-ECFCs (scale bar: 100 μm). **b** Co-immunofluorescence staining to detect Ki-67 (a proliferation marker, red) and nor-ECFCs, hypo-ECFCs, *si-STAT3*/hypo-ECFCs, or scramble siRNA/hypo-ECFCs (human nuclear antigen (HNA)-positive cells, green). DAPI (blue) was used for nuclear staining (scale bar: 50 μm). **c** Bar graph shows the results of number of PCNA^+^ cells 3 days after transplantation. **d** Quantitative analysis of Ki-67/HNA/DAPI triple-positive cells 3 days after transplantation of nor-ECFCs, hypo-ECFCs, *si-STAT3*/hypo-ECFCs, or scramble siRNA/hypo-ECFCs. **P < 0.01 vs. nor-ECFCs; ^##^ *P* < 0.01 vs. hypo-ECFCs; ^$$^ *P* < 0.01 vs. si-*STAT3*/hypo-ECFC. *ECFC* endothelial colony-forming cell, *PBS* phosphate-buffered saline

In the previous in vitro experiments, we demonstrated that the culture of ECFCs in hypoxic conditions activated the STAT3 signaling pathway and increased their proliferative potential. We hypothesized that hypoxic-preconditioned culturing of ECFCs (hypo-ECFCs) would be beneficial for repairing the damaged tissue in the hindlimb ischemia injury model by providing cells with a better proliferative rate to the site of ischemic injury. To evaluate the in vivo regulation of the STAT3 pathway, we transplanted nor-ECFCs and hypo-ECFCs into nude mice with hindlimb ischemia. After 3 days, ischemic tissues transplanted with hypo-ECFCs exhibited significantly higher levels of STAT3 activation than tissues transplanted with nor-ECFCs (Fig. 3a). Next, the distribution of STAT3 in hypo-ECFCs was evaluated. Hypo-ECFCs exhibited an increase in the number of phospho-STAT3/HNA/DAPI-positive cells in the ECFC population (Fig. 3b). Accordingly, more proliferating cell nuclear antigen (PCNA)-positive cells were found in tissue transplanted with hypo-ECFCs than that transplanted with nor-ECFCs (Fig. 4a, c). HNA and proliferating cell marker (Ki-67)-double positive cells were more abundant at 3 days in tissue receiving hypo-ECFCs than that receiving nor-ECFCs (Fig. 4b, d). However, STAT*3*-specific siRNA-transfected hypo-ECFCs (*si-STAT3*/hypo-ECFCs) displayed poor proliferation in ischemic tissue.

### STAT3 upregulates BCL3 expression and promotes ECFC survival during ischemic stress

**Fig. 5:**
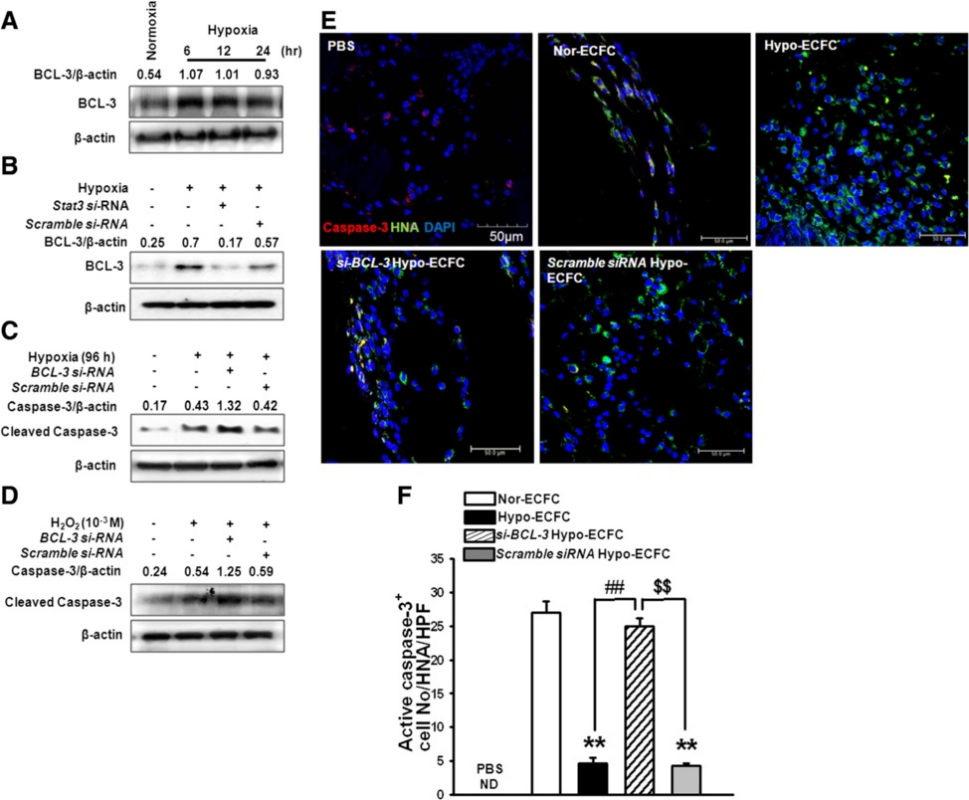
Modulation of the STAT3-BCL3 axis enhances survival induced by ischemic stress. **a** Endothelial colony-forming cells (ECFCs) were exposed to hypoxia for various times (0–24 h). BCL3 expression was detected by western blot analysis. **b** ECFCs were transfected with *STAT3*-siRNA for 48 h prior to exposure to hypoxic conditions for 24 h. BCL3 was detected by western blot analysis. **c** ECFCs were transfected with *BCL3*-siRNA for 48 h prior to exposure to hypoxic conditions for 96 h. Cleaved caspase-3 was detected by western blot analysis. **d** ECFCs were transfected with *BCL3*-siRNA for 48 h prior to treatment with H_2_O_2_ (10^−3^ M) for 8 h followed by western blot analysis for cleaved caspase-3. **e** Co-immunofluorescence staining was used to detect apoptosis (caspase-3, an apoptosis marker, red) and nor-ECFCs, hypo-ECFCs, si-*BCL3/*hypo-ECFCs, or scramble siRNA/hypo-ECFCs (human nuclear antigen (HNA)-positive cells, green). DAPI (blue) was used for nuclear staining. Arrows indicate caspase-3^+^/HNA^+^/DAPI^+^ cells (scale bar: 50 μm). **f** Quantitative analysis of caspase-3/HNA/DAPI triple-positive cells conducted 3 days after transplantation of nor-ECFCs, hypo-ECFCs, si-*BCL3*/hypo-ECFCs, or scramble siRNA/hypo-ECFCs. **P < 0.01 vs. nor-ECFCs; ^##^ *P* < 0.01 vs. hypo-ECFCs; ^$$^ *P* < 0.01 vs. si-*STAT3*/hypo-ECFC; n = 10

Next, we determined whether hypoxia-induced activation of STAT3 led to the activation of BCL3, a known target of STAT3 [28], subsequently promoting cell survival [17]. Normoxic cells were found to express a very low level of BCL3, whereas hypoxic cells exhibited elevated BCL3 expression (Fig. 5a). We examined whether STAT3 was involved in the regulation of BCL3 expression. As expected, the hypoxia-induced increase in BCL3 expression was attenuated by *STAT3*-specific siRNAs (Fig. 5b). Additionally, siRNA-targeting *BCL3* in long-term hypoxic conditions (96 h) in the presence of a high concentration of H_2_O_2_ (10^−3^ M) increased cleaved caspase-3 levels, a marker for apoptosis, relative to hypoxia alone (Fig. 5c, d). Furthermore, immunofluorescence staining for caspase-3 and HNA in ischemic muscle at 3 days after transplantation revealed that apoptotic ECFCs were significantly reduced after transplantation with hypo-ECFCs compared to after transplantation with nor-ECFCs, but *BCL3*-specific siRNA-transfected hypo-ECFCs (*si-BCL3*/hypo-ECFCs) displayed poor survival in ischemic tissue (Fig. 5e, f). These data suggest that STAT3 increases cell survival and prevents apoptosis via the induction of BCL3.

### STAT3 promotes upregulation of VEGF secretion in ischemic tissue

**Fig. 6:**
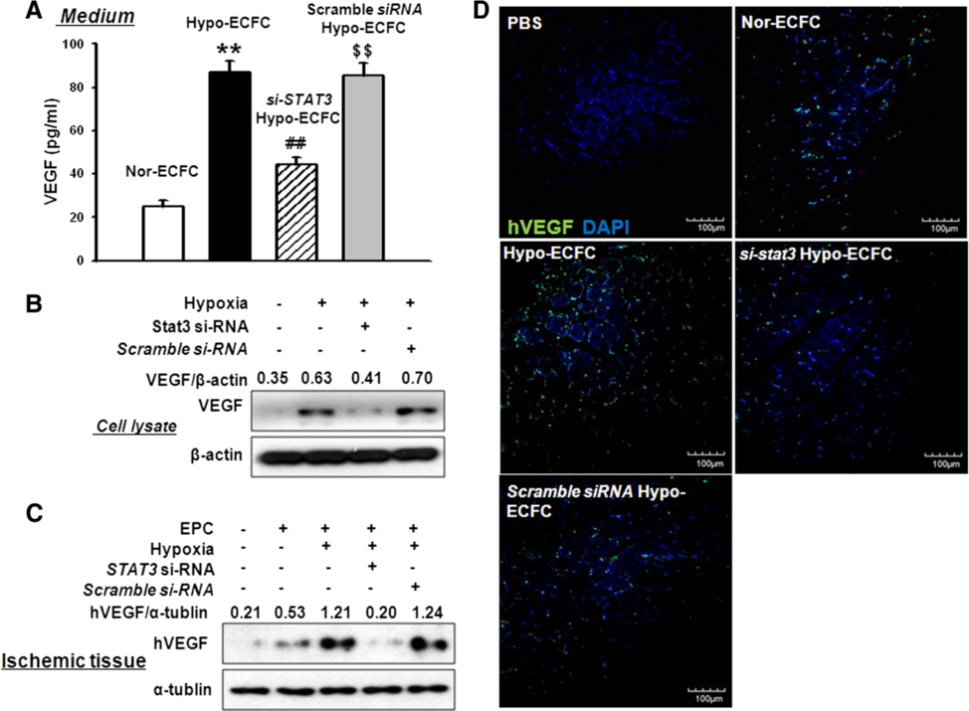
Transplanted hypo-ECFCs enhance secretion of vascular endothelial growth factor (VEGF) in ischemic limb muscle via STAT3 signaling. **a, b** ECFCs, si-*STAT3*-ECFCs, and scramble siRNA hypo-ECFCs were cultured in normoxic or hypoxic conditions for 12 h, and VEGF levels were determined by using ELISA and western blot analysis. The results are expressed as the mean ± SD. **c** Western blotting analyses of VEGF conducted 3 days after transplantation of nor-ECFCs, hypo-ECFCs, si-*STAT3/*hypo-ECFCs, or scramble siRNA/hypo-ECFCs. **d** Immunofluorescence staining for VEGF in ischemic tissues conducted 3 days after transplantation of nor-ECFCs, hypo-ECFCs, si-*STAT3/*hypo-ECFCs, or scramble siRNA/hypo-ECFCs (n = 7). **P < 0.01 vs. nor-ECFCs; ^##^ *P* < 0.01 vs. hypo-ECFCs; ^$$^ *P* < 0.01 vs. si-*STAT3*/hypo-ECFC. *ECFC* endothelial colony-forming cell

Transplantation of ECFCs into ischemic tissue enhances the paracrine secretion of angiogenic growth factors such as VEGF. To determine whether VEGF was secreted by hypo-ECFCs, we analyzed the secretion and expression of VEGF by ELISA and western blot analysis. Hypo-ECFCs clearly exhibited higher secretion and expression of VEGF than nor-ECFCs (Fig. 6a, b). To evaluate whether si-*STAT3/*hypo-ECFCs alter the secretion of VEGF in ischemic tissue, we transplanted hypo-ECFCs, nor-ECFCs, and si-*STAT3/*hypo-ECFCs into the hindlimb ischemic injury site. Western blot analysis revealed that VEGF expression within this tissue was elevated after hypo-ECFC transplantation, while VEGF expression following transplantation with *si-STAT3/*hypo-ECFCs or nor-ECFCs was only slightly higher than that of the PBS-treated control (Fig. 6c). Moreover, VEGF expression was detected by immunofluorescence staining in hypo-ECFCs at 3 days after transplantation, whereas minimal VEGF expression was detected in tissue transplanted with *si-STAT3/*hypo-ECFCs (Fig. 6d). These results suggested that hypo-ECFCs were able to induce the secretion of VEGF, and that the activation of STAT3 was responsible for VEGF induction.

### Hypoxic preconditioning of ECFCs promotes vascular repair in ischemic limbs

**Fig. 7:**
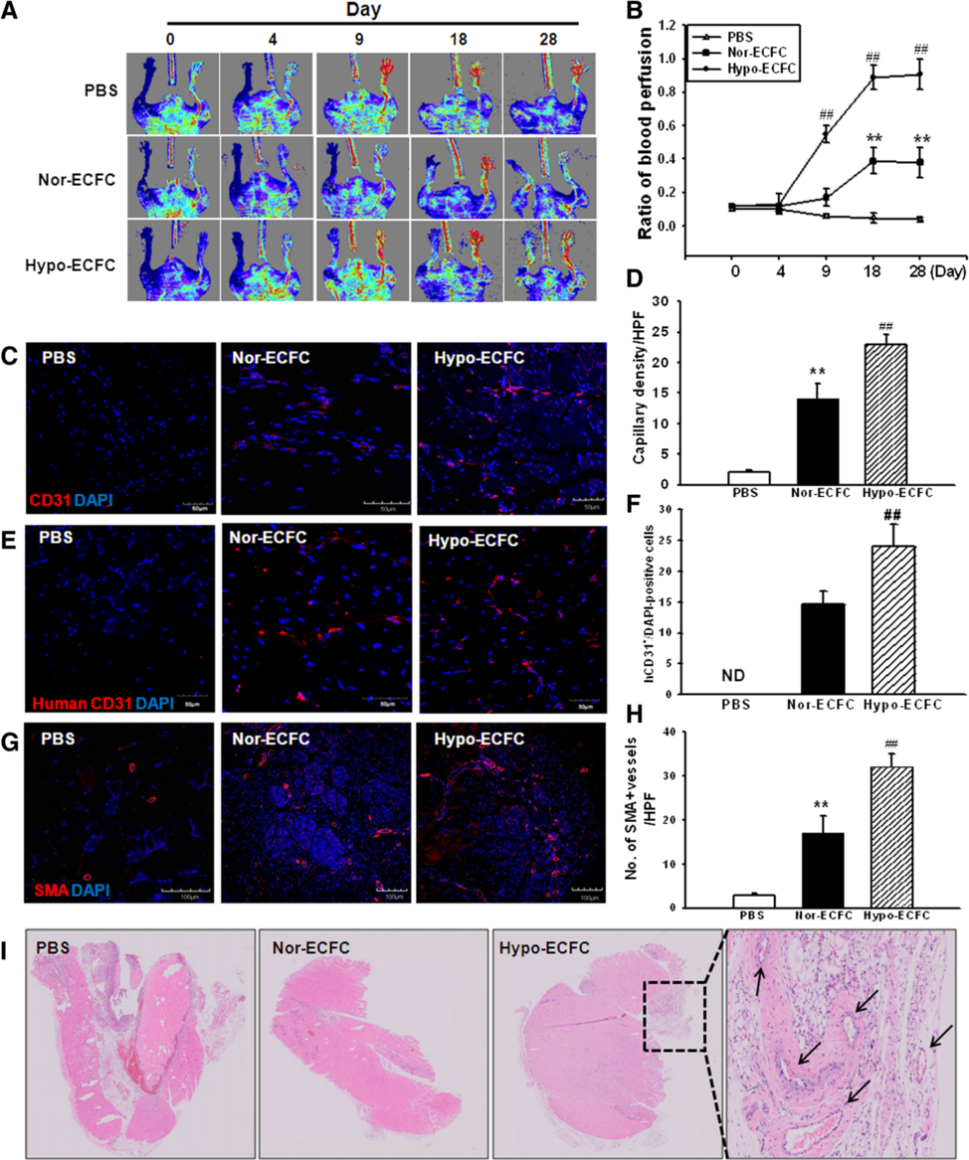
Hypoxic preconditioning of endothelial colony-forming cells (ECFCs) enhances functional recovery after limb ischemia. **a** Improvements in blood flow recovery were evaluated using LDPI analysis in the ischemic limbs of 8-week-old Balb/C nude mice injected with phosphate-buffered saline (PBS), nor-ECFCs, and hypo-ECFCs at 0, 4, 9, 18, and 28 days post-surgery. **b** The ratio of blood perfusion (blood flow of the left ischemic limb/blood flow of the right non-ischemic limb) was measured in the three groups. Values represent means ± SD. **P < 0.01 vs. PBS group; ^##^ *P* < 0.01 vs. nor-ECFCs. **c**, **e** Representative images of mouse-specific CD31^+^ and human-specific CD31^+^ tissue at 28 days after transplantation of nor-ECFC or hypo-ECFC into hindlimb ischemia (scale bar: 50 μm; n = 10). **g** Representative images of arteriole structures (α-SMA staining for arterioles, red fluorescence)-positive tissue at 28 days after transplantation of nor-ECFC or hypo-ECFC into hindlimb ischemia (scale bar: 50 μm; n = 10). Quantification (**d**, **f**, **h**) represents cell numbers analyzed per high-power field (HPF). Values represent means ± SD. **P < 0.01 vs. nor-ECFCs; ^##^ *P* < 0.01 vs. hypo-ECFCs. **i** H&E staining was used to produce histological images of tissue 28 days after transplantation of nor-ECFCs or hypo-ECFCs into hindlimb ischemia

To examine whether hypo-ECFCs would promote neovascularization in vivo, a hindlimb ischemia mouse model was used and analyzed with LDPI. Ischemia was induced in the mouse hindlimb, and equal numbers of ECFCs were injected into the ischemic sites. Subsequent laser Doppler perfusion measurements revealed that the blood flow in the ischemic right hindlimb was lower than that in the control left hindlimb. The hypo-ECFC treatment group displayed increased hindlimb perfusion compared with the nor-ECFC group (Fig. 7a, b). Transplantation of hypo-ECFCs promoted angiogenesis within ischemic tissue. Immunofluorescence staining for CD31 and quantification of capillary density was significantly enhanced after transplantation with hypo-ECFC compared to transplantation with nor-ECFCs (Fig. 7c, d). To elucidate that transplanted human ECFCs were involved in vessel formation, immunofluorescent staining for human-specific CD31 was performed. Human-specific CD31-positive cells were more abundant in animals 28 days after treatment with hypo-ECFCs than in those treated with nor-ECFCs (Fig. 7e, f). Similarly, immunofluorescence staining for α-SMA showed that arteriole formation was enhanced by transplanting hypo-ECFC (Fig. 7g, h). H&E staining showed that blood flow increased with transplantation of hypo-ECFCs compared with the blood flow observed following transplantation of nor-ECFCs (Fig. 7i).

## Discussion

In the present study, we demonstrated that hypoxia increased the proliferative, clonogenic, and survival potential of ECFCs via JAK2/STAT3 phosphorylation and STAT3-induced expression of BCL3. Although resident human EPCs have been reported to contribute significantly to vascular repair at sites of ischemia and are capable of differentiating into endothelial cells [24–26], the development of a protocol that exploits the advantages of culture methods to improve the quality and quantity of EPCs for clinical applications is necessary. There is accumulating evidence that modulation of retroviral transduction of the human telomerase gene [29] and the combination of growth factors in serum-free culture [30] can enhance the progenitor properties of EPCs. All of these methods, however, result in genetic modification or the enrichment of cells with oncogenic potential [31]. The protocol presented herein takes advantage of the specific ability of EPCs to survive under hypoxia without modification that could lead to any aberrant genetic and biological alterations [32]. Based on their normal morphology and intact genetic integrity, as well as the enhancement of their angiogenic potential upon transplantation, the protocol presented herein is safe to use for the preparation of ECFCs for clinical applications. To our knowledge, this study is the first to demonstrate that hypoxia improves the functionality of ECFCs. These effects resulted in the functional activation of ECFCs by hypoxia, thereby facilitating ECFC-mediated neovascularization and functional recovery in ischemic disease.

Previous reports have demonstrated that STAT3 contributes to the hypoxia-induced enhancement of self-renewal in embryonic stem cells [33, 34] and other adult stem cells [35, 36], as well as hypoxia-induced angiogenesis [37], hypoxia preconditioned human EPC-induced neovascularization [32], and tumor vasculature [38]. In the present study, consistent with these previous reports, hypoxic culture of ECFCs increased stem cell surface markers (CD34, c-Kit, and CXCR4) and VEGFR2, and augmented the proliferative and clonogenic potential of ECFCs by enhancement of VEGF expression through the modulation of the STAT3 signaling pathway. However, in the study by Hofmann et al. [39], ECFC function decreased in response to hypoxic conditions (1 % O_2_), and this ECFC bioactivity was rescued by co-culture with MSC/progenitor cells. This discrepancy in response of hypoxia on ECFC could be explained by differences in the oxygen concentration and duration of hypoxia. Although hypoxic culture conditions of at least 2 % are indispensable to robust human ESC clone recovery and self-renewal and multipotency of ESCs, a long-term, severe hypoxic condition resulted in cell damage and apoptosis [40, 41]. In addition, we previously reported that the hypoxic preconditioning increased proliferation of human EPCs through augmentation of S phase and cell cycle regulatory proteins expression [41]. Furthermore, Ingram et al. [23] identified high proliferative potential endothelial colony-forming cells (HPP-ECFCs) in HUCB. The increased proliferative and clonogenic potential of ECFCs suggests that hypoxic preconditioning induces augmentation of the HPP-ECFC population via the STAT3-BCL3 circuit. Importantly, we focused on BCL3 expression by the regulation of STAT3. BCL3 was increased by phosphorylation of STAT3 and facilitated ECFC survival in vitro and in vivo. BCL3 is a proto-oncogene that belongs to the IκB family, and its aberrant expression was first reported in chronic B cell lymphocytic leukemia [42]. Elevated levels of BCL3 have been detected in a large number of cancers, including breast cancer [14], and it has been reported that BCL3 can have an anti-apoptotic effect in cancer [28]. Although several studies have examined the function and regulation of BCL3, little is known about the mechanism of BCL3 in physiological and pathophysiological conditions, especially in stem cells. Our findings revealed that BCL3 was upregulated under hypoxic conditions, resulting in the prevention of apoptosis induced by ischemic stress both in vitro and in vivo. The present study demonstrates, for the first time, that BCL3 could play a pivotal role in proliferation, clonogenic potential, and survival of ECFCs by the direct regulation of hypoxia-induced STAT3.

ECFCs and their therapeutic capabilities have been explored in previous studies. Although ECFCs have been shown to differentiate into endothelial cells in vivo [26, 43–45], the leading concept is that ECFCs mediate tissue repair through the secretion of angiogenic factors. Some evidence suggests that hypoxia may enhance the secretion of angiogenic factors from ECFCs, thus indicating another possible mechanism by which hypoxic preconditioning enhances the tissue-repair potential of ECFCs. ECFCs have been demonstrated to secrete multiple proangiogenic factors, including VEGF, FGF, and placental growth factor, in response to hypoxic stimulation [46]. In this report, we show that the expression of human VEGF was higher in hypo-ECFCs than in nor-ECFCs. In the mouse hindlimb ischemia model, transplanted hypo-ECFCs exhibited higher STAT3 activity increased human VEGF expression, while sequentially transplanted si-*STAT3*-transduced hypo-ECFCs displayed decreased VEGF expression, which is indicative of ongoing angiogenesis, as late as 4 weeks after ECFC transplantation. In addition, mice injected with hypo-ECFCs exhibited enhanced neovascularization. These findings, in conjunction with the upregulation of angiogenic cytokines, are consistent with the known vasculogenic mechanisms of ECFCs. Our findings demonstrate the potential clinical applicability of hypoxia-stimulated ECFCs as a novel therapeutic neovascularization strategy in ischemic diseases.

In this study, we investigated ex vivo culture conditions for the enhancement of the proliferative, clonogenic potential and prevention of ischemic stress-induced apoptosis in human ECFCs. The optimized ex vivo culture conditions consequently promoted robust cell survival and rapid functional cellular repair in a hindlimb ischemia cell transplantation model. The techniques and strategies developed here will be useful in determining the requirements of transplanted human ECFCs for in vivo survival, recruitment to areas with hypoxic damage, and the initiation of endogenous repair. As a result of these studies, we will gain a better understanding of how injected stem cells are recruited to damaged tissue to enhance revascularization by endogenous cells.

## Conclusions

**Fig. 8:**
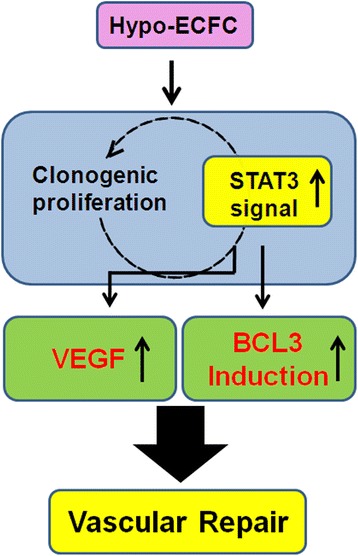
Schematic representation of proposed mechanisms by which hypoxia increases clonogenic and proliferative potential, and enhances hypo-ECFC-mediated neovasculogenesis. Hypoxic preconditioning increases the clonogenic and proliferative potential of endothelial colony-forming cells (ECFCs) via the STAT3 pathway and augments the survival of ECFCs via STAT3-mediated BCL3 and vascular endothelial growth factor (VEGF) expression. These effects enhance ECFC-mediated neovasculogenesis in ischemic diseases

To our knowledge, this study shows for the first time that hypo-ECFCs enhance the recovery of cells from vascular injury by modulating the STAT3/BCL3 axis via VEGF expression (Fig. 8). The therapeutic efficiency of ECFCs can thus be controlled by modifying the culture conditions or activation of the STAT3/BCL3 pathway, providing insights into the bioactivity circuit needed for ECFC therapy that will improve efficiency in clinical applications.

## Abbreviations

α-SMA: Alpha-smooth muscle actin
BCL3: B cell lymphoma 3
bFGF: Basic fibroblast growth factor
BM: Bone marrow
DAPI: 4′,6-diamidino-2-phenylindole phenylindole
EBM: Endothelial basal media
ECFC: Endothelial colony-forming cell
ECL: Enhanced chemiluminescence
EGF: Epidermal growth factor
ELISA: Enzyme-linked immunosorbent assay
EPC: Endothelial progenitor cell
FACS: Fluorescence-activated cell sorter
FBS: Fetal bovine serum
H&E: Hematoxylin and eosin
HIF-1α: Hypoxia inducible factor-1α
HNA: Human nuclear antigen
HPP-ECFC: High proliferative potential endothelial colony-forming cell
HUCB: Human umbilical cord blood
IGF-1: Insulin-like growth factor-1
JAK2: Janus kinase 2
LDPI: Laser Doppler perfusion imaging
MNC: Mononuclear cell
MSC: Mesenchymal stem cell
PBS: Phosphate-buffered saline
PCNA: Proliferating cell nuclear antigen
siRNA: Small interfering RNA
STAT3: Signal transducer and activator of transcription
VEGF: Vascular endothelial growth factor
vWF: von Willebrand factor

## References

[1] Asahara T, Murohara T, Sullivan A, Silver M, van der Zee R, Li T (1997). Isolation of putative progenitor endothelial cells for angiogenesis. Science.

[2] Yoo SY, Kwon SM (2013). Angiogenesis and its therapeutic opportunities. Mediators Inflamm.

[3] Kalka C, Masuda H, Takahashi T, Kalka-Moll WM, Silver M, Kearney M (2000). Transplantation of ex vivo expanded endothelial progenitor cells for therapeutic neovascularization. Proc Natl Acad Sci U S A.

[4] Asahara T, Masuda H, Takahashi T, Kalka C, Pastore C, Silver M (1999). Bone marrow origin of endothelial progenitor cells responsible for postnatal vasculogenesis in physiological and pathological neovascularization. Circ Res.

[5] Taguchi A, Soma T, Tanaka H, Kanda T, Nishimura H, Yoshikawa H (2004). Administration of CD34^+^ cells after stroke enhances neurogenesis via angiogenesis in a mouse model. J Clin Invest.

[6] Zhao ZM, Li HJ, Liu HY, Lu SH, Yang RC, Zhang QJ (2004). Intraspinal transplantation of CD34^+^ human umbilical cord blood cells after spinal cord hemisection injury improves functional recovery in adult rats. Cell Transplant.

[7] Semenza GL (1998). Hypoxia-inducible factor 1: master regulator of O_2_ homeostasis. Curr Opin Genet Dev.

[8] Stoeltzing O, McCarty MF, Wey JS, Fan F, Liu W, Belcheva A (2004). Role of hypoxia-inducible factor 1α in gastric cancer cell growth, angiogenesis, and vessel maturation. J Natl Cancer Inst.

[9] Haque N, Rahman MT, Abu Kasim NH, Alabsi AM (2013). Hypoxic culture conditions as a solution for mesenchymal stem cell based regenerative therapy. Sci World J.

[10] Jung JE, Lee HG, Cho IH, Chung DH, Yoon SH, Yang YM (2005). STAT3 is a potential modulator of HIF-1-mediated VEGF expression in human renal carcinoma cells. FASEB J.

[11] Pawlus MR, Wang L, Murakami A, Dai G, Hu CJ (2013). STAT3 or USF2 contributes to HIF target gene specificity. PLoS One.

[12] Choi HJ, Lee JM, Kim H, Nam HJ, Shin HJ, Kim D (2010). Bcl3-dependent stabilization of CtBP1 is crucial for the inhibition of apoptosis and tumor progression in breast cancer. Biochem Biophys Res Commun.

[13] Thornburg NJ, Pathmanathan R, Raab-Traub N (2003). Activation of nuclear factor-κB p50 homodimer/Bcl-3 complexes in nasopharyngeal carcinoma. Cancer Res.

[14] Cogswell PC, Guttridge DC, Funkhouser WK, Baldwin AS (2000). Selective activation of NF-κ B subunits in human breast cancer: potential roles for NF-κ B2/p52 and for Bcl-3. Oncogene.

[15] Parrinello S, Samper E, Krtolica A, Goldstein J, Melov S, Campisi J (2003). Oxygen sensitivity severely limits the replicative lifespan of murine fibroblasts. Nat Cell Biol.

[16] Ceradini DJ, Kulkarni AR, Callaghan MJ, Tepper OM, Bastidas N, Kleinman ME (2004). Progenitor cell trafficking is regulated by hypoxic gradients through HIF-1 induction of SDF-1. Nat Med.

[17] Liu X, Wu X, Cai L, Tang C, Su J (2003). Hypoxic preconditioning of cardiomyocytes and cardioprotection: phophorylation of HIF-1α induced by p42/p44 mitogen-activated protein kinases is involved. Pathophysiology.

[18] Grayson WL, Zhao F, Izadpanah R, Bunnell B, Ma T (2006). Effects of hypoxia on human mesenchymal stem cell expansion and plasticity in 3D constructs. J Cell Physiol.

[19] Grayson WL, Zhao F, Bunnell B, Ma T (2007). Hypoxia enhances proliferation and tissue formation of human mesenchymal stem cells. Biochem Biophys Res Commun.

[20] Hung SC, Pochampally RR, Hsu SC, Sanchez C, Chen SC, Spees J (2007). Short-term exposure of multipotent stromal cells to low oxygen increases their expression of CX3CR1 and CXCR4 and their engraftment in vivo. PLoS One.

[21] Potier E, Ferreira E, Andriamanalijaona R, Pujol JP, Oudina K, Logeart-Avramoglou D (2007). Hypoxia affects mesenchymal stromal cell osteogenic differentiation and angiogenic factor expression. Bone.

[22] http://www.orient.co.kr. Lab animals company web site.

[23] Ingram DA, Mead LE, Tanaka H, Meade V, Fenoglio A, Mortell K (2004). Identification of a novel hierarchy of endothelial progenitor cells using human peripheral and umbilical cord blood. Blood.

[24] Lee MO, Song SH, Jung S, Hur S, Asahara T, Kim H (2012). Effect of ionizing radiation induced damage of endothelial progenitor cells in vascular regeneration. Arterioscler Thromb Vasc Biol.

[25] Hur J, Yoon CH, Kim HS, Choi JH, Kang HJ, Hwang KK (2004). Characterization of two types of endothelial progenitor cells and their different contributions to neovasculogenesis. Arterioscler Thromb Vasc Biol.

[26] Lee SH, Lee JH, Yoo SY, Hur J, Kim HS, Kwon SM (2013). Hypoxia inhibits cellular senescence to restore the therapeutic potential of old human endothelial progenitor cells via the hypoxia-inducible factor-1α-TWIST-p21 axis. Arterioscler Thromb Vasc Biol.

[27] Kim HJ, Lee HJ, Jun JI, Oh Y, Choi SG, Kim H (2009). Intracellular cleavage of osteopontin by caspase-8 modulates hypoxia/reoxygenation cell death through p53. Proc Natl Acad Sci U S A.

[28] Ahmed SU, Milner J (2009). Basal cancer cell survival involves JNK2 suppression of a novel JNK1/c-Jun/Bcl-3 apoptotic network. PLoS One.

[29] Murasawa S, Llevadot J, Silver M, Isner JM, Losordo DW, Asahara T (2002). Constitutive human telomerase reverse transcriptase expression enhances regenerative properties of endothelial progenitor cells. Circulation.

[30] Thum T, Bauersachs J (2005). Endothelial progenitor cells as potential drug targets. Curr Drug Targets Cardiovasc Haematol Disord.

[31] Serakinci N, Guldberg P, Burns JS, Abdallah B, Schrodder H, Jensen T (2004). Adult human mesenchymal stem cell as a target for neoplastic transformation. Oncogene.

[32] Akita T, Murohara T, Ikeda H, Sasaki K, Shimada T, Egami K (2003). Hypoxic preconditioning augments efficacy of human endothelial progenitor cells for therapeutic neovascularization. Lab Investig.

[33] Barbosa HS, Fernandes TG, Dias TP, Diogo MM, Cabral JM (2012). New insights into the mechanisms of embryonic stem cell self-renewal under hypoxia: a multifactorial analysis approach. PLoS One.

[34] Cartwright P, McLean C, Sheppard A, Rivett D, Jones K, Dalton S (2005). LIF/STAT3 controls ES cell self-renewal and pluripotency by a Myc-dependent mechanism. Development.

[35] Carcamo-Orive I, Tejados N, Delgado J, Gaztelumendi A, Otaegui D, Lang V (2008). ERK2 protein regulates the proliferation of human mesenchymal stem cells without affecting their mobilization and differentiation potential. Exp Cell Res.

[36] Tu B, Du L, Fan QM, Tang Z, Tang TT (2012). STAT3 activation by IL-6 from mesenchymal stem cells promotes the proliferation and metastasis of osteosarcoma. Cancer Lett.

[37] Kang SH, Yu MO, Park KJ, Chi SG, Park DH, Chung YG (2010). Activated STAT3 regulates hypoxia-induced angiogenesis and cell migration in human glioblastoma. Neurosurgery.

[38] Martinive P, Defresne F, Bouzin C, Saliez J, Lair F, Gregoire V (2006). Preconditioning of the tumor vasculature and tumor cells by intermittent hypoxia: implications for anticancer therapies. Cancer Res.

[39] Hofmann NA, Ortner A, Jacamo RO, Reinisch A, Schallmoser K, Rohban R (2012). Oxygen sensing mesenchymal progenitors promote neo-vasculogenesis in a humanized mouse model in vivo. PLoS One.

[40] Forsyth NR, Musio A, Vezzoni P, Simpson AH, Noble BS, McWhir J (2006). Physiologic oxygen enhances human embryonic stem cell clonal recovery and reduces chromosomal abnormalities. Cloning Stem Cells.

[41] Lee SH, Lee YJ, Han HJ (2011). Role of hypoxia-induced fibronectin-integrin β1 expression in embryonic stem cell proliferation and migration: Involvement of PI3K/Akt and FAK. J Cell Physiol.

[42] Ohno H, Takimoto G, McKeithan TW (1990). The candidate proto-oncogene bcl-3 is related to genes implicated in cell lineage determination and cell cycle control. Cell.

[43] Lee JH, Lee SH, Yoo SY, Asahara T, Kwon SM (2013). CD34 hybrid cells promote endothelial colony-forming cell bioactivity and therapeutic potential for ischemic diseases. Arterioscler Thromb Vasc Biol.

[44] Lee SH, Lee KB, Lee JH, Kang S, Kim HG, Asahara T (2014). Selective interference targeting of lnk in umbilical cord-derived late endothelial progenitor cells improves vascular repair, following hindlimb ischemic injury, via regulation of JAK2/STAT3 signaling. Stem Cells.

[45] Cho JG, Lee JH, Hong SH, Lee HN, Kim CM, Kim SY (2015). Tauroursodeoxycholic acid, a bile acid, promotes blood vessel repair by recruiting vasculogenic progenitor cells. Stem Cells.

[46] Prabhu S, Ignatova A, Park ST, Sun XH (1997). Regulation of the expression of cyclin-dependent kinase inhibitor p21 by E2A and Id proteins. Mol Cell Biol.

